# Recurrence in Paraesophageal Hernia: Patient Factors and Composite Surgical Repair in 862 Cases

**DOI:** 10.1007/s11605-023-05856-w

**Published:** 2023-11-14

**Authors:** Chu Luan Nguyen, David Tovmassian, Michael Zhou, Doruk Seyfi, Anna Isaacs, Suzanna Gooley, Gregory L. Falk

**Affiliations:** 1 Department of Upper Gastrointestinal Surgery, Concord Repatriation General Hospital, Concord, NSW 2139 Australia; 2 Department of Surgery, The University of Sydney, Camperdown, NSW 2050 Australia; 3 Sydney Heartburn Clinic, Lindfield, NSW 2070 Australia

**Keywords:** Paraesophageal hiatal hernia, Fundoplication, Recurrence

## Abstract

**Background:**

Repair of giant paraesophageal hernia (PEH) is associated with a considerable hernia recurrence rate by objective measures. This study analyzed a large series of laparoscopic giant PEH repair to determine factors associated with anatomical recurrence.

**Method:**

Data was extracted from a single-surgeon prospective database of laparoscopic repair of giant PEH from 1991 to 2021. Upper endoscopy was performed within 12 months postoperatively and selectively thereafter. Any supra-diaphragmatic stomach was defined as anatomical recurrence. Patient and hernia characteristics and technical operative factors, including “composite repair” (360° fundoplication with esophagopexy and cardiopexy to right crus), were evaluated with univariate and multivariate analysis.

**Results:**

Laparoscopic primary repair was performed in 862 patients. The anatomical recurrence rate was 27.3% with median follow-up of 33 months (IQR 16, 68). Recurrence was symptomatic in 45% of cases and 29% of these underwent a revision operation. Hernia recurrence was associated with younger age, adversely affected quality of life, and were associated with non-composite repair. Multivariate analysis identified age < 70 years, presence of Barrett’s esophagus, absence of “composite repair”, and hiatus closure under tension as independent factors associated with recurrence (HR 1.27, 95%CI 0.88–1.82, *p* = 0.01; HR 1.58, 95%CI 1.12–2.23, *p* = 0.009; HR 1.72, 95%CI 1.2–2.44, *p* = 0.002; HR 2.05, 95%CI 1.33–3.17, *p* = 0.001, respectively).

**Conclusion:**

Repair of giant PEH is associated with substantial anatomical recurrence associated with patient and technique factors. Patient factors included age < 70 years, Barrett’s esophagus, and hiatus tension. “Composite repair” was associated with lower recurrence rate.

**Supplementary Information:**

The online version contains supplementary material available at 10.1007/s11605-023-05856-w.

## Introduction

The prediction and prevention of hernia recurrence after paraesophageal hernia (PEH) repair may improve symptoms after surgery. Continued effort in attempts to reduce recurrence rate is therefore indicated. Giant PEH has been defined variably with some defining it as greater than 30% of herniated stomach through the diaphragmatic hiatus, and others as herniation of greater than 50% ^1^. These herniae are often symptomatic and also have a risk of strangulation, gastric necrosis, and perforation. The operation for giant PEH is technically complex as more mediastinal dissection and longer operation duration is required, and occurs in elderly comorbid patients with attendant risks ^2^.

Although symptom resolution after repair of hiatus herniae can be achieved in most patients ^3^, reported anatomical recurrence rates remain high, ranging from 42 to 66% ^4–6^. It was previously thought that most patients with small recurrent hernia were asymptomatic, but recent reports suggest that recurrence of any size may cause symptoms ^7–9^. Reduced quality of life (QOL) in patients with recurrent hernia has also been recently demonstrated ^10^. Therefore, refinement of hiatus hernia repair and prevention of recurrence of any size remains desirable. Anatomical recurrence can be used as a measure for comparison of surgical techniques as endeavours are made to improve outcomes ^4–6^.

Whilst repair of small symptomatic hiatus herniae has been well described, there is a paucity of data regarding factors that influence rates of anatomical recurrence after laparoscopic repair of giant PEH ^11–13^. Understanding patient and hernia factors predictive of recurrence could aid patient selection and optimization of modifiable risk factors. Knowledge of operative factors associated with recurrence may provide potential for modification and refinement of surgical technique. The aim of this study was to determine patient, hernia, and operative factors associated with anatomical recurrence after laparoscopic giant PEH repair in a large series.

## Methods

### Study Design and Patients

Data was obtained from a prospectively maintained database. An earlier study of this data focused on trends in outcomes over time, including morbidity and mortality, while this current study evaluated factors predictive of hernia recurrence, both of which are too substantial to be reported in one study ^14^. Patients with giant PEH who underwent primary laparoscopic non-mesh giant PEH repair, performed between 1991 and 2021, were evaluated. Operations were predominantly performed by the senior author (GLF) or post fellowship senior trainees under direct supervision. Indications for surgery included symptomatic giant PEHs, including those with sub-acute obstructive symptoms or volvulus, significant gastro-esophageal symptoms, or dyspnoea.

Inclusion criteria for this study were elective and semi-elective repair of symptomatic giant PEH, defined as ≥ 30% of herniated stomach through the diaphragmatic hiatus based on intraoperative findings, and type III or IV hernias. Exclusion criteria were open hernia repairs, use of mesh during a short locum period, Collis gastroplasty used for a short period during an unpublished randomized study, revisional operation, primary open surgery, and < 12 months of follow-up (Appendix 1).

### Surgical Technique

All patients underwent laparoscopic repair of giant PEH without mesh and the “composite repair” group underwent cardio-esophageal junction fixation. Overweight patients were vigorously encouraged to lose weight if body mass index (BMI) > 30. Principles of repair were complete reduction of hernia sac, thoracic dissection of esophagus, vagal preservation, crural closure with deep non-absorbable sutures over sizing bougie, and 360° fundoplication fixed to the cardio-esophageal junction. Initially the fundoplication was similar to that described by DeMeester ^15^, and Rossetti and Hell ^16^. A “composite repair”, which incorporated an esophagopexy and cardiopexy to the right crus pillars, was utilized after a policy change from 2007 in an attempt to reduce recurrence rates ^17^.

**Fig. 1:**
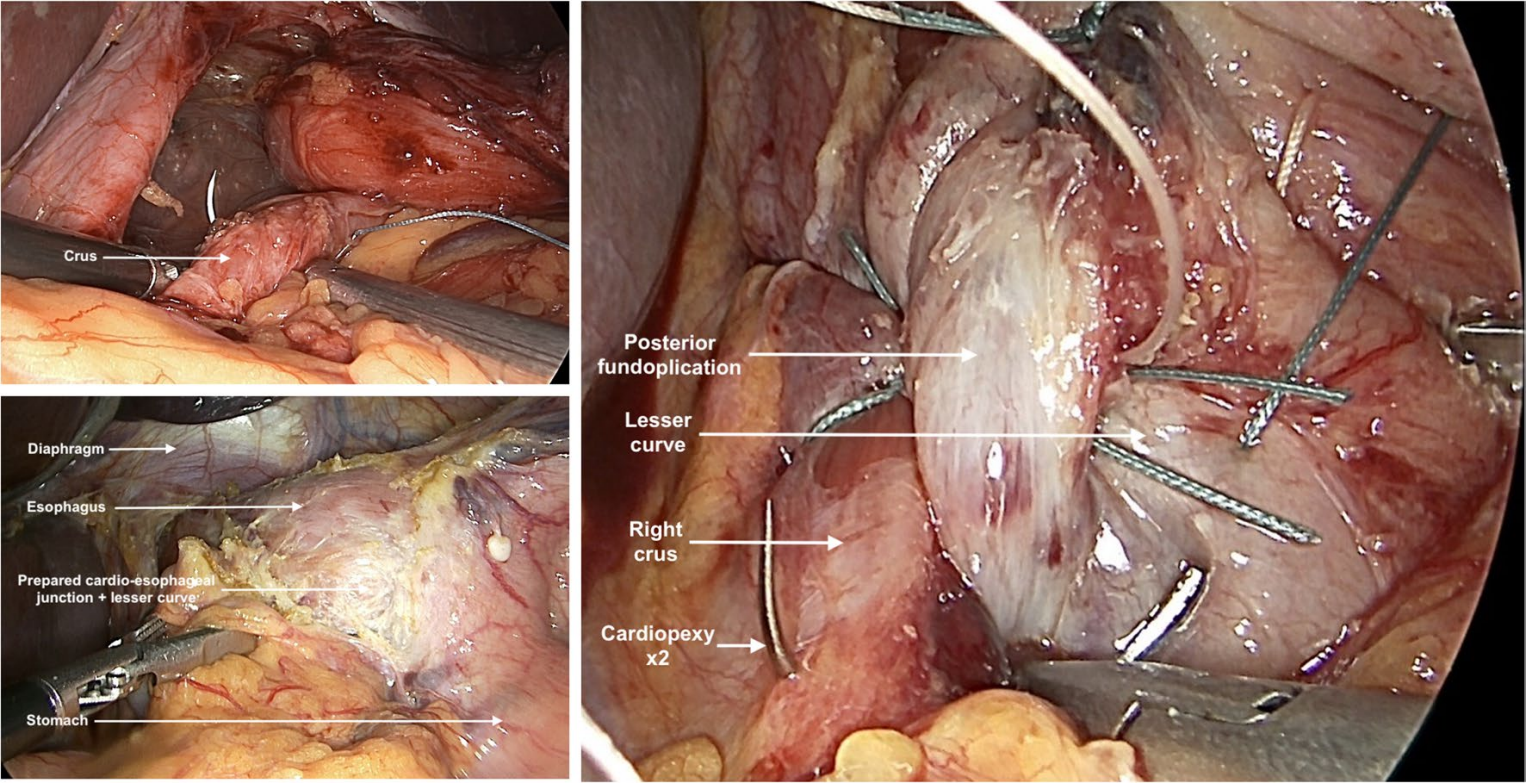
Operative photo of “composite repair” of a giant paraesophageal hernia. Top left image: Hiatus repair with deep crural suture. Bottom left image: Preparation of lesser curve and mobilized anterior vagus nerve. Right image: Cardiopexy with suture through lesser curve, posterior fundoplication, and right crus

Repair of the hiatus was performed by placement of two to four posterior sutures and anterior hiatus sutures deeply to pick up fascia and tendinous core of the left and right crura. Surgical mesh repair was not used. For the fixation and fundoplication, the superior aspect of the posterior cardiopexy was passed around the esophagus, aiming to create a soft wrap with no tension on the stomach or esophagus. Two sutures incorporating esophagus and cardio-esophageal junction, posterior fundus, median arcuate ligament, and repaired crus were placed. Total fundoplication was completed with two sutures through the left-sided anterior fundus, posterior fundus, and right crus. Further sutures were placed attaching left fundus to posterior left crus. Generally, a 56 French bougie in males or a 52 French bougie in smaller females was used to check diaphragmatic and fundoplication tightness under direct vision during the procedure (Fig. 1) ^17^.

### Outcome Variables

**Fig. 2:**
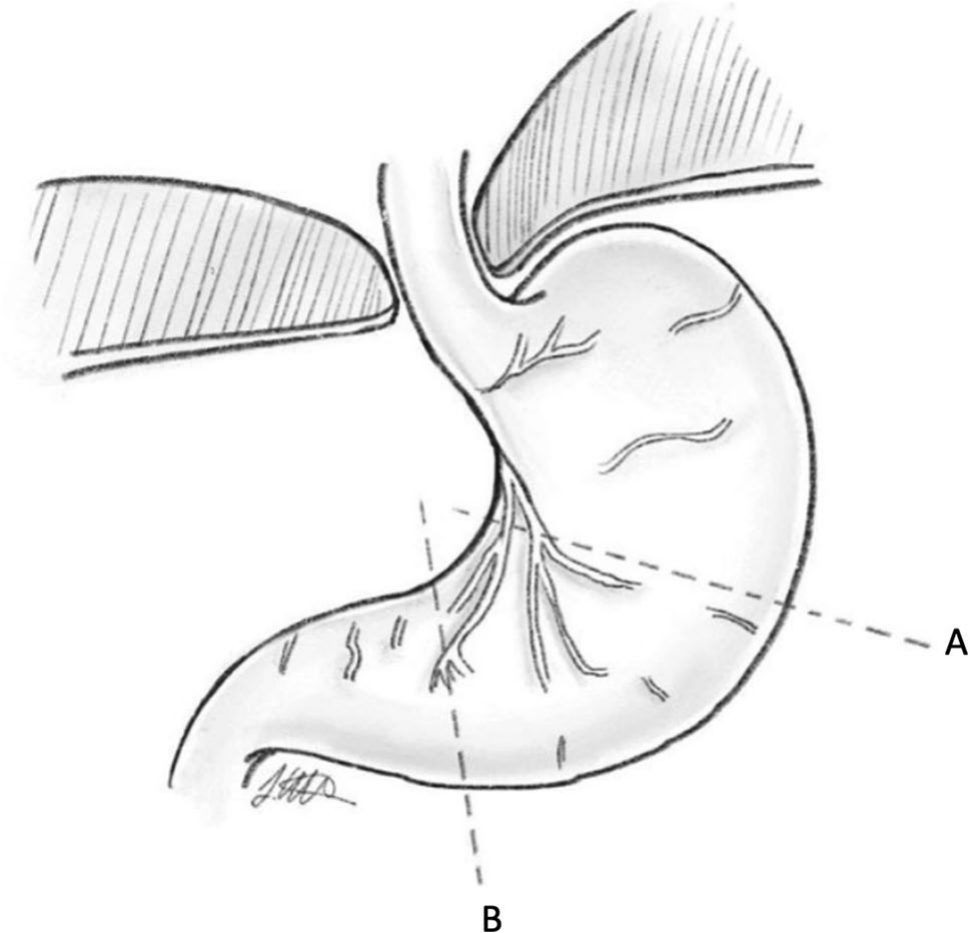
Hernia size estimation by intraoperative assessment expressed by percentage of stomach in mediastinum. Stomach depicted below the diaphragm. Line B depicts 75% and line A depicts 50%. Landmarks used at laparoscopy were the pylorus (100% herniation), “crow’s foot” of the incisura (75%), and a point halfway between the crow’s foot and angle of His (50%) at the level of the anterior arch of the hiatus

Patient factors included the following: age, gender, BMI at surgery, ASA (American Society of Anesthesiologists) grade, and preoperative and postoperative symptoms including QOL surveys. Hernia characteristics included the following: hernia type, hernia size, and hiatus size. Hiatus size was characterised visually, and number of stiches for repair considered a surrogate measure. Operative factors included those relevant to hiatal closure: “composite repair” use, number of sutures (anterior, posterior, and total sutures) in the hiatal repair, whether there was perceived tension on the hiatus during closure, and length of intra-abdominal esophagus achieved (< 2cm or ≥ 2cm). Hernia size was estimated by intraoperative assessment and expressed by the percentage of stomach in the mediastinum (Fig. 2). Hiatus size was classified by surgical estimation as “moderately large”, “large”, or “very large”. Tension was assessed as pressure required for adequate apposition of crural pillars by the senior author (GLF) calibrated by bougienage. Postoperative outcomes included the following: complications graded according to Clavien-Dindo Classification, results of upper endoscopy or barium swallow to detect anatomical recurrence, symptom recurrence, and reinterventions or reoperations. Anatomical recurrence was defined as evidence of any supra-diaphragmatic stomach of either < 2cm or > 2cm of intrathoracic stomach, measured vertically from the anterior hiatus.

### Follow-up

**Fig. 3:**
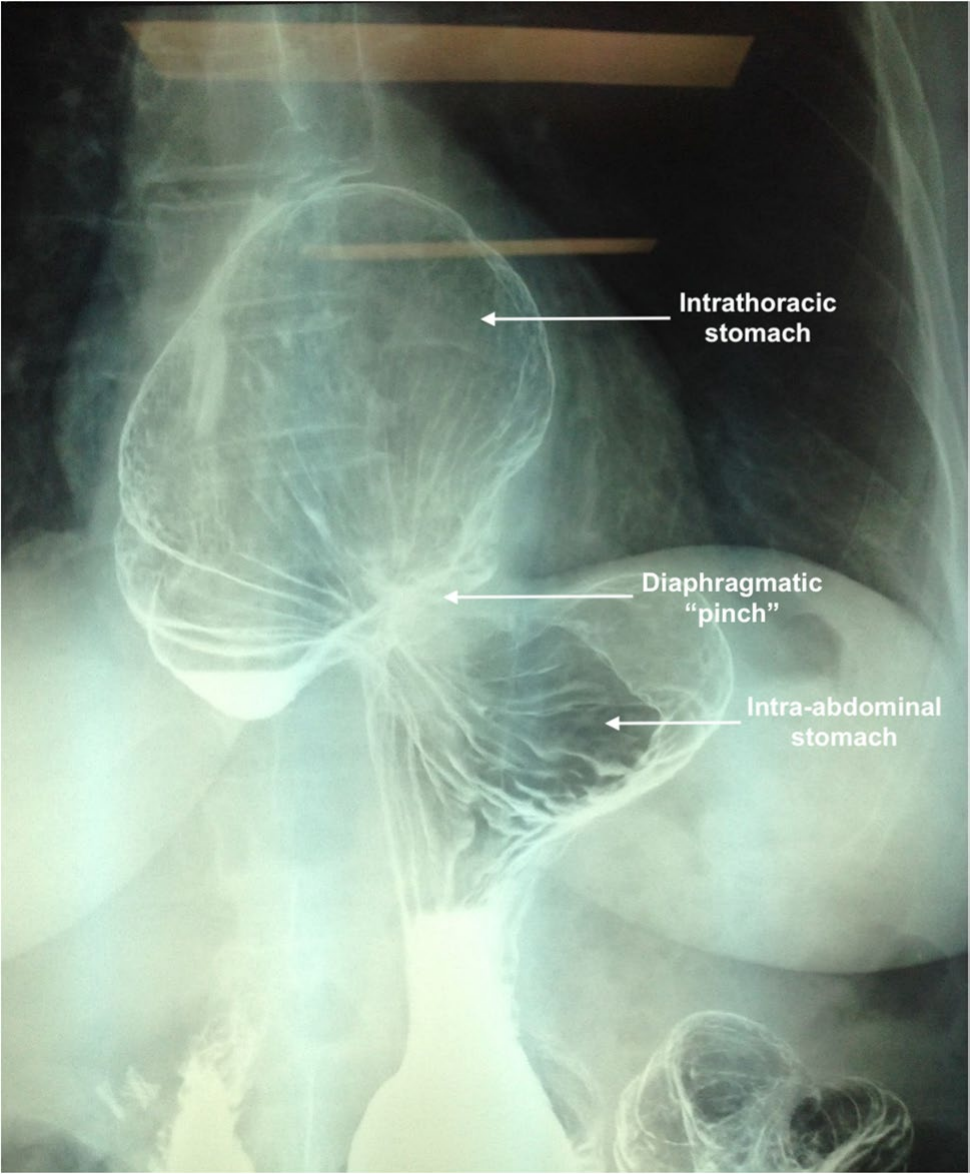
Barium swallow exam demonstrating a recurrent giant hiatus hernia following hiatus hernia repair

Follow-up with upper endoscopy was performed at least once within the first postoperative year and was then planned 2–3 years subsequently. The presence of a recurrent hernia, esophagitis on upper endoscopy, and Barrett’s esophagus on histopathology was recorded. A barium meal was performed where endoscopy was impractical (Fig. 3). QOL questionnaires were self-administered preoperative, and at 12 months postoperative using the Gastrointestinal Quality of Life Index (GIQOLI), which assesses gastrointestinal-specific health-related QOL. Total score ranges from 0 to 144 with higher scores suggesting better QOL ^18^.

### Statistical Analysis

Data was presented as median with interquartile range (IQR) or mean with standard deviation (SD) when appropriate. Time to hernia recurrence and median follow-up were analyzed using Kaplan–Meier methods. Univariate analysis was performed using two-tailed Student’s *t* test or Wilcoxon rank-sum test for continuous variables, and chi-squared or Fisher’s exact test for categorical variables. Covariates were analyzed with the Cox proportional hazards model. Multivariate regression was performed if *p* ≤ 0.2 on univariate regression. Statistical significance was considered as *p* < 0.05. Statistical analysis was performed with RStudio, version 2022.07.2.

## Results

There was a total of 862 laparoscopic non-mesh repair of giant PEHs over the 30-year study period. The median age was 70 years (IQR 63, 77), with a mean BMI of 27.9 (SD 4.3) and median ASA of 3 (IQR 2–3). Most hernia were type III (87.4%), with median hernia size of 66% (IQR 45, 80), and the majority had “large” hiatus size (76%). The majority (97.6%) of patients underwent a Nissen 360° fundoplication wrap while 19 (2.2%) had a Dor anterior 270° wrap and two (0.23%) had a Toupe posterior wrap. Median follow-up was 33 months (IQR 16, 68). The rate of anatomical hernia recurrence was 27.4%. The symptom recurrence rate was 21%. Median time to anatomical hernia recurrence was 26 months (IQR 10, 59). The rate of revision surgery for symptomatic hernia recurrence was 3.8% (33 patients) (Appendix 2).

### Characteristics of Cohorts with and Without Recurrence

The characteristics of patients with and without anatomical hernia recurrence after repair were mostly similar in terms of patient variables of mean BMI, gender, median ASA, and preoperative symptoms; hernia variables of hernia type, hernia size, and hiatus size; and operative variables related to repair including intra-abdominal esophageal length, number of crural sutures used, and proportion with hiatal closure under tension (Table 1).

**Table 1:** Characteristics of patients with and without anatomical recurrence after laparoscopic repair of giant paraesophageal hernia

	Recurrence cohort (*n* = 236)	Non-recurrence cohort (*n* = 626)	*p* value
Patient variables			
Age, median (IQR)	68 (61, 76)	70 (64, 77)	**0.012**
BMI, mean (SD)	28 (4.1)	27.9 (4.5)	0.733
Gender, *N* (%)			0.966
Male	80 (33.9%)	215 (34.3%)	
Female	156 (66.1%)	411 (65.7%)	
ASA, median (IQR)	3 (2, 3)	3 (2, 3)	0.669
Barrett’s esophagus, *N* (%)			** < 0.001**
Present	67 (28.4%)	107 (17.1%)	
Absent	169 (71.6%)	519 (82.9%)	
Preoperative symptoms, *N* (%)			
Dyspnoea	154 (65.3%)	414 (66.1%)	0.172
Pain	120 (50.8%)	333 (53.2%)	0.916
Heartburn	123 (52.1%)	330 (52.7%)	1
Regurgitation	122 (51.7%)	313 (50%)	0.342
Dysphagia	125 (53%)	310 (49.5%)	0.127
Cough	66 (28%)	165 (26.4%)	0.062
Hernia variables			
Hernia type, N (%)			0.419
II	23 (9.7%)	46 (7.3%)	
III	204 (86.4%)	549 (87.7%)	
IV	9 (3.8%)	31 (5%)	
Hernia size^a^, median % (IQR)	70 (40, 80)	70 (40, 80)	0.843
Hiatus size, N (%)			0.204
Moderate	13 (5.5%)	39 (6.2%)	
Large	189 (80.1%)	466 (74.4%)	
Very large	34 (14.4%)	121 (19.3%)	
Operative variables			
“Composite repair”^b^			** < 0.001**
Present	118 (50%)	413 (66%)	
Absent	118 (50%)	213 (44%)	
Esophageal length^c^			0.094
< 2cm	34 (15.4%)	63 (10.8%)	
≥ 2cm	187 (84.6%)	522 (89.2%)	
Anterior crural repair^d^			0.198
Present	186 (78.8%)	518 (82.7%)	
Absent	50 (21.2%)	108 (17.3%)	
Total crural sutures, median (IQR)	3 (3, 4)	4 (3, 4)	0.38
Anterior crural sutures	1 (1, 2)	1 (1, 2)	0.17
Posterior crural sutures	2 (2, 3)	2 (2, 3)	0.14
Hiatus closure under tension, *N* (%)			0.079
Present	30 (12.7%)	53 (8.5%)	
Absent	206 (87.3%)	573 (91.5%)	

*BMI* body mass index, *ASA* American Society of Anesthesiologists, *SD* standard deviation, *IQR* interquartile range

^a^Percentage in mediastinum

^b^Incorporated 360° fundoplication with esophagopexy and cardiopexy to right crus

^c^Intra-abdominal esophageal length

^d^At least one anterior crural suture used

Patients with recurrence were younger compared to those without (median 68 versus 70 years, respectively, *p* = 0.012), and had a higher proportion of Barrett’s esophagus on preoperative upper endoscopy (28.4% versus 17.1%, respectively, *p* < 0.001). “Composite repair” was utilized less frequently in those with recurrent hernia (50% versus 66%, respectively, *p* < 0.001) (Table 1).

### Outcomes of Cohorts with and Without Recurrence

Anatomical hernia recurrence occurred in 236 (27.4%) patients. One hundred six (44.9%) of these patients were symptomatic, and 33 (29%) of these symptomatic patients underwent a revision operation (all revisions underwent a laparoscopic repair, and 24 of the revisions had a composite repair at index operation). The symptoms prompting revision included dysphagia, reflux, and pain, with endoscopic or radiological evidence of hernia recurrence. Ninety-seven (41.1%) recurrences were larger than 2 cm in size, while 139 (58.9%) were less than 2 cm in size. A similar proportion of patients with large and small recurrences were symptomatic (43.9% versus 44.4%, respectively, *p* = 0.986). Seventy-five (8.7%) patients with symptom recurrence and without anatomical hernia recurrence were managed medically (Table 2).

**Table 2:** Outcomes of patients with and without anatomical recurrence after laparoscopic repair of giant paraesophageal hernia

	Recurrence cohort (*n* = 236)	Non-recurrence cohort (*n* = 626)	*p* value
Hernia recurrence size, *N* (%)			
> 2cm	97 (41.1%)		
< 2cm	139 (58.9%)		
Symptom recurrence, *N* (%)			
Present	106 (44.9%)	75 (12%)	**< 0.001**
Absent	130 (55.1%)	551 (88%)	
Months to recurrence, median (IQR)	26 (10, 59)		
Follow-up, median months (IQR)	35 (16, 55.2)	42 (18.8, 80.2)	0.19
Postoperative endoscopy, *N* (%)			
Dilatation	6 (2.5%)	24 (3.8%)	0.342
Esophagitis	29 (12.3%)	20 (3.2%)	**< 0.001**
Complication^a^	3 (1.3%)	7 (1.1%)	1
Revision surgery	33 (14%)	0	**< 0.001**
QOL GIQLI score^b^, mean (SD)			
Preoperative	66.2 (43.9)	78 (37.3)	**0.014**
At 12 months postoperative	104.5 (24.8)	111.3 (22.8)	0.091

*IQR* interquartile range

^a^Clavien-Dindo grade III and above

^b^GIQLI, Gastrointestinal Quality of Life Index score (max score of 144)

There were no significant differences in number of Clavien-Dindo grade ≥ III complications between the recurrence and non-recurrence cohorts. Patients with recurrent hernia had a higher proportion of esophagitis on postoperative upper endoscopy (12.3% versus 3.2%, respectively, *p* < 0.001). There were no significant differences in the proportion of patients requiring endoscopic dilatation (Table 2).

Preoperative mean QOL scores using GIQLI were reduced in both recurrence and non-recurrence cohorts when compared to a perfect score. Patients without recurrence had significantly better preoperative scores compared to patients in the recurrence group (78 versus 66.2, respectively, *p* = 0.014). There were no significant differences in postoperative QOL scores between the two cohorts and both cohorts achieved improvement in scores reported at 12 months postoperative (Table 2). Survey participation rate was 80.5% (694/862).

### Factors Associated with Anatomical Hernia Recurrence

Univariate analysis of anatomical hernia recurrence did not significantly correlate with preoperative symptoms, gender, ASA grade, hernia size, hernia type, or total crural sutures used. By multivariate analysis, age < 70 years, presence of Barrett’s esophagus preoperative, absence of “composite repair”, and hiatus closure under tension were significantly associated with anatomical hernia recurrence (HR 1.27, 95%CI 0.88–1.82, *p* = 0.01; HR 1.58, 95%CI 1.12–2.23, *p* = 0.009; HR 1.72, 95%CI 1.2–2.44, *p* = 0.002; HR 2.05, 95%CI 1.33–3.17, *p* = 0.001, respectively) (Table 3).

**Table 3:** Multivariate analysis of predictors associated with anatomical recurrence after laparoscopic repair of giant paraesophageal hernia

Variable	Hazard ratio (95% CI)	*p* value
Age		**0.01**
≥ 70	1.00	
< 70	1.27 (0.88, 1.82)	
BMI		0.175
< 30	1.00	
≥ 30	1.24 (0.91, 1.68)	
Barrett’s esophagus		**0.009**
Absent	1.00	
Present	1.58 (1.12, 2.23)	
Hiatus size “very large”		0.39
Absent	1.00	
Present	1.2 (0.78, 1.85)	
“Composite repair”^a^		**0.002**
Present	1.00	
Absent	1.72 (1.2, 2.44)	
Esophageal length^b^		0.55
< 2cm	1.00	
≥ 2cm	0.86 (0.54, 1.39)	
Anterior crural repair^c^		0.672
Absent	1.00	
Present	0.89 (0.52, 1.52)	
Posterior crural sutures		0.442
≤ 3	1.00	
> 3	1.15 (0.81, 1.63)	
Hiatus closure under tension		**0.001**
Absent	1.00	
Present	2.05 (1.33, 3.17)	

*BMI* body mass index, *CI* confidence interval

^a^Incorporated 360° fundoplication with esophagopexy and cardiopexy to right crus

^b^Intra-abdominal esophageal length

^c^At least one anterior crural suture used

There was a significant correlation between hiatus size and hiatus closure under tension. A significantly higher proportion of patients with “very large” hiatus size underwent hiatus closure under tension, compared to patients with “large” or “moderate” hiatus size (19.4% versus 7.6% versus 5.8%, respectively, *p* < 0.001).

### “Composite Repair” or Standard Repair

**Fig. 4:**
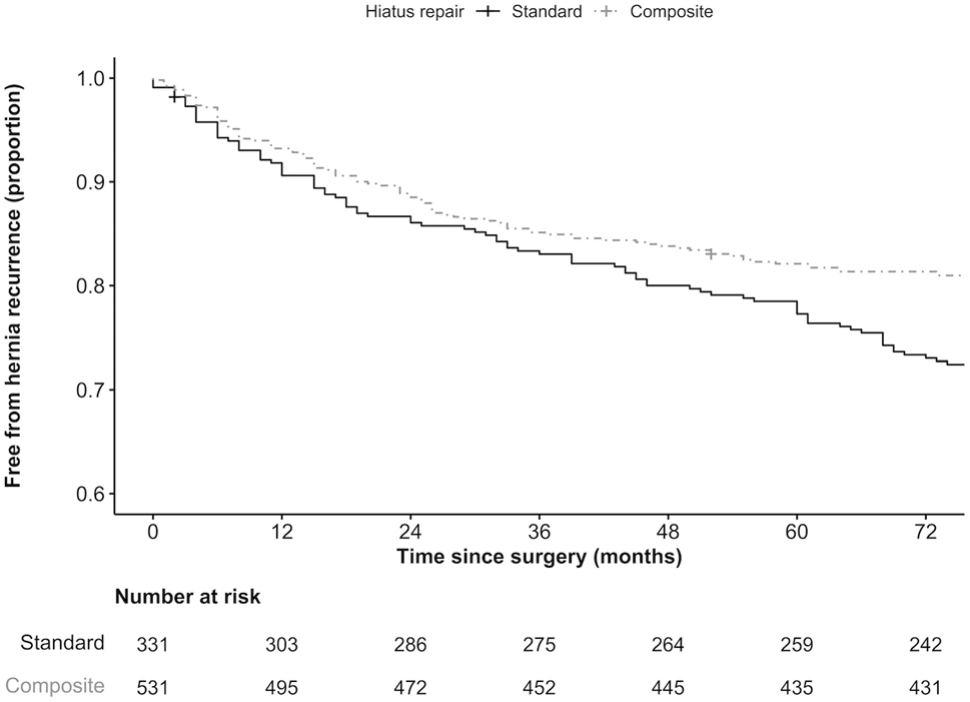
Kaplan–Meier curves of comparative freedom from anatomical hernia recurrence over 6 years stratified by standard and “composite” repair (*p* < 0.001)

There were 531 (61.6%) patients that underwent “composite repair” compared to 331 (38.4%) that underwent surgery without. The patient demographics and hernia characteristics were mostly similar. Median follow-up for patients that underwent “composite repair” was less compared to those that did not (24 months [IQR 15, 38.5] versus 45 months [IQR 20, 71], respectively, *p* < 0.001). Analysis of recurrence in those patients with and without composite repair using Kaplan–Meier method showed a significant (*p* < 0.001) reduction in anatomical hernia recurrence at comparable time periods, suggesting a positive effect of “composite repair” (Fig. 4).

## Discussion

There is a paucity of data on the predictors of hernia recurrence following laparoscopic repair of hiatus hernia despite recurrence rates having been extensively evaluated ^6,7, 11, 12, 19–23^. Data on the prediction and potential prevention of hernia recurrence after repair is important for the ongoing refinement of this operation to improve durability of repair. Small numbers of reports have suggested that obesity, hernia size and type, technical issues including suturing under tension, inadequate esophageal mobilizsation, and tension, as well as postoperative retching and vomiting, were risk factors for recurrence ^11,12, 21^. Some of these factors were similarly found to contribute to recurrence in this study. These included age < 70 years, presence of Barrett’s esophagus, and hiatus closure under tension. Absence of “composite repair” was also a significant predictor of recurrence and unique to this study.

It has been established that laparoscopic repair yields high rates of symptom resolution and improved QOL ^11,24^. When evaluated objectively with imaging, however, there can be high rates of anatomical recurrence of up to 66% ^8^. There is evidence that the high prevalence of anatomical recurrence following repair does not directly correlate with symptomatic recurrence ^4–6^. The anatomical recurrence rate in this study (27.4%) is fairly low considering the liberal definition of recurrence used. Less than half of the patients with anatomical recurrence in this series were symptomatic, and less than a third of those with symptoms required revision surgery, consistent with the low rates described in the literature ^22,25–27^. Recurrent hernia of < 2cm or > 2cm in size were also found to be similarly associated with recurrent symptoms in this series, suggesting that patients can be symptomatic even from small recurrences. These findings indicate room for potential improvement in the durability of laparoscopic repair of giant PEH when using anatomical recurrence as a measure for durability of repair.

Factors associated with recurrence, particularly for giant PEH, have yet to be fully defined. Risk factors for recurrence following repair would theoretically be those that stress the diaphragm, increase the abdominal to thoracic cavity pressure gradient, or make it difficult for hiatus tissue to resist forces applied to it ^28^. Age less than 70 years was associated with higher risk of recurrence in this study. Age was not a significant predictor of recurrence after laparoscopic repair of large type III hiatus hernia and giant PEH in other studies ^12,13, 25, 26^. Recurrence may not be solely related to patient age, but it is possible younger patients are more likely to have increased activity level and potential of higher generation of intra-abdominal pressure so stressing the repair. Similar to previous studies, BMI and ASA grade were not found to significantly influence rates of recurrence in this study ^13^. A deficiency in understanding the effect of BMI in this study is the recording of weight after significant preoperative instructed weight loss. Perhaps weight at initial presentation may have been predictive, as could postoperative weight gain but this data was not prospectively stored.

Presence of Barrett’s esophagus preoperative was identified to be an independent factor associated with hernia recurrence. Patients with severe esophagitis or Barrett’s esophagus have been demonstrated to have a significantly higher prevalence of larger hiatus hernia than patients with non-erosive reflux disease or mild esophagitis ^29^. Higher recurrence rates following anti-reflux surgery have been shown to occur in Barrett’s esophagus compared to uncomplicated gastro-esophageal disease ^30^. This could reflect lack of pliability in the esophagus secondary to more severe and prolonged acid exposure.

The hernia type and size in this study did not significantly influence recurrence rates, similar to previous studies ^13^. Hernia size as a predictor of recurrence has had conflicting results in studies evaluating repair of hiatus hernia of any size. Larger hernia size (> 5cm) was a significant predictor of recurrence in some studies ^25,27, 31^ and not significant in others ^13,26^. Studies reporting a centimetre measure are largely reporting the elevation of the lower esophageal sphincter above the crural diaphragm, not true hernia size (amount of stomach volume in the mediastinum) and could be misleading.

Given that few patient or hernia related factors have been shown to be associated with recurrence, the more pertinent factors associated with recurrence could arguably be operative factors. This provides room for improvement of technical aspects of the operation for better outcomes. The generally agreed upon essential technical aspects of hiatus hernia repair include sac removal from mediastinum, esophageal mediastinal mobilization to allow a tension free intra-abdominal esophageal length, avoidance of sutures under too much tension at the crural closure, and fundoplication for the anti-reflux barrier ^17,23, 32^. A non-mesh approach yielded reasonable results in this series. Crural repair augmentation by mesh hernioplasty is controversial and not yet definitively proven to be superior to sutured hiatal closure ^17^. Recent meta-analyses have demonstrated that both techniques deliver comparable outcomes ^19,33–36^.

Collis gastroplasty used to be an aspect of anti-reflux surgery and hiatus hernia repair when peptic strictures were more prevalent which caused “short esophagus” ^37^. The reported incidence of short esophagus in published series of PEH ranges widely from 2 to 80% ^26,38^. The use of Collis gastroplasty also ranges widely with some units reporting prevalence of up to 66% ^7,11, 39^. An esophageal length < 2cm was uncommon in this current study (12%) and Collis gastroplasty was largely abandoned throughout this series which is a similar trend to other reports ^11,40^. Short esophagus in PEH probably was associated with lesser mediastinal dissection as the rate has diminished over time. It was not found to be significantly associated with hernia recurrence in this series as other factors such as absence of “composite repair” and hiatus closure under tension were more significant. The concept of short esophagus may not be as critical as once thought, given that fairly low recurrence rates were achieved in this study largely without Collis gastroplasty use, which is similar to other contemporary series, suggesting surgical technique improved over time ^11,40^.

Most surgeons place sutures primarily in the posterior hiatus and behind the esophagus when closing the diaphragmatic hiatus. It has been observed in studies, including a video analysis of over 100 reoperations for hiatus hernia repairs that recurrences most often occur in the anterior hiatus ^28,41^. Recurrences through the anterior hiatus are thought to be secondary to dilatation, rather than disruption or failure of the hiatal suture line ^28^. Deficiency of the central tendon may be the primary mechanism for anterior crural defects as they have been shown to increase over time ^42^. “Telescoping” of the cardio-esophageal junction through a dilated hiatus and fundoplication, frequently referred to as “slipped wrap”, could possibly be reduced with a “composite repair”. This approach, involving fixation of cardio-esophageal junction to the right crus along with the fundoplication, was shown to significantly reduce the risk of hernia recurrence in this series.

Hiatus closure under tension was identified as a significant risk factor for recurrence in this series. This is potentially a critical aspect of the repair if hiatus dilatation is one of the main mechanisms of recurrence. The technical aspects of achieving a low tension hiatus repair and calibration of the hiatus varies between surgeons ^17,43^. The number of anterior, posterior, and total crural sutures were not significant predictors of recurrence in this study. It would seem the number of sutures in hiatal repair was not an adequate surrogate of hiatal tension, which was predictive of increased recurrence rates. Perception of hiatal tension however could be used to adapt technique once the risk factor is identified.

### Limitations

Strengths of this study include the large number of patients with giant PEH from a single institution, the prospective nature of the data, with postoperative follow-up consisting of both objective and subjective data. It is limited by missing data, particularly with intra-abdominal esophageal lengths and QOL surveys, which may introduce bias into the analysis. A more detailed description of the type of recurrence to include information such as the proportion of “slipped wraps” was not possible due to incomplete data. There was no “control” preoperative endoscopy or barium swallow available for all the patients. All patients had total reduction of stomach at surgery. In this way, all hernias detected on postoperative endoscopy or barium swallow were therefore recurrent. Some, however, may have recurred before objective review. The follow-up time is also not uniform, with symptomatic patients arguably more likely to return for review compared with patients who remained asymptomatic which may falsely degrade results. Follow-up was significantly shorter, as expected, in the “composite repair” group as those patients were operated later in the study period.

### Conclusion

The multifactorial nature of anatomical recurrence makes it a complex issue to prevent. Although laparoscopic repair of giant PEH is safe and effective for symptom relief, it remains a complex operation with room for further refinement when anatomical recurrence is used as a measure. Age < 70 years and Barrett’s esophagus were associated with recurrence. Knowledge of these preoperative patient-related factors could help with patient selection, advice, and informed decision-making. Surgeons could have more control over prevention of recurrence by correct application of established operative-related factors including adequate intra-abdominal esophageal length, minimal tension crural closure, fundoplication, and potentially placement of anterior hiatal suture(s) cognisant of the possible anterior dilatation of the hiatus. Use of a “composite repair” could be added to this armamentum. Refinement of operative factors for this operation is an ongoing process, but this current approach with laparoscopic non-mesh repair has yielded non-inferior results.

### Supplementary Information

Below is the link to the electronic supplementary material.

Supplementary file1 (DOCX 89 KB)

## Data Availability

The data that support the findings of this study are available on request from the corresponding author, GLF.
