# Supplementary material for: Recurrence in Paraesophageal Hernia: Patient Factors and Composite Surgical Repair in 862 Cases
Source: J Gastrointest Surg. 2023 Nov 14;27(12):2733–42. doi: 10.1007/s11605-023-05856-w (PMC10837213; doi:10.1007/s11605-023-05856-w)
Supplement: Supplementary file 1 — Supplementary file1 (DOCX 89 KB) [file 11605_2023_5856_MOESM1_ESM.docx]

**SUPPLEMENTARY MATERIAL**

Appendix 1. Selection criteria for laparoscopic non-mesh repair of giant paraesophageal hernia study


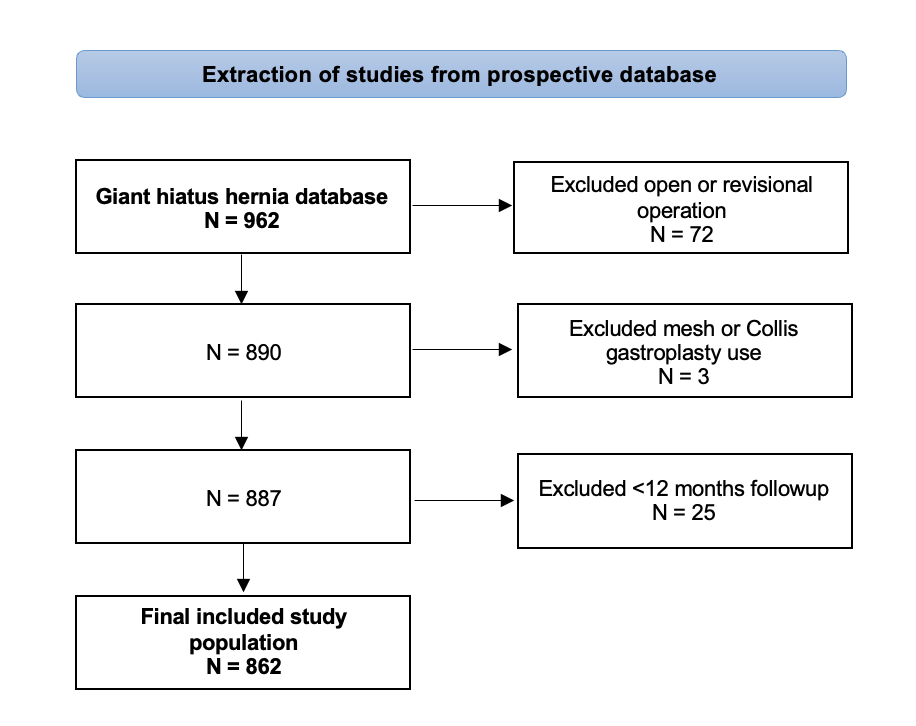


Appendix 2. Characteristics of patients undergoing laparoscopic repair of giant paraoesophageal hernia

|  | **Whole cohort,**  **N = 862** |
| --- | --- |
| **Patient characteristics** |  |
| Age, median (IQR) | 70 (63, 77) |
| Gender, N (%) |  |
| Female | 567 (65.8%) |
| Male | 295 (34.2%) |
| BMI, mean (SD) | 27.9 (4.3) |
| ASA, median (IQR) | 3 (2-3) |
| Barrett’s esophagus, N (%) | 174 (20.2%) |
| Symptoms, N (%) |  |
| Dyspnoea | 568 (65.9%) |
| Chest/ epigastric pain | 453 (52.6%) |
| Heartburn | 453 (52.6%) |
| Regurgitation | 435 (50.5%) |
| Dysphagia | 435 (50.5%) |
| Cough | 231 (26.8%) |
| **Hernia characteristics** |  |
| Hernia type, N (%) |  |
| II | 69 (8%) |
| III | 753 (87.4%) |
| IV | 40 (4.6%) |
| Hernia size^a^, median % (IQR) | 66 (45, 80) |
| Hiatus size, N (%) |  |
| Moderate | 52 (6%) |
| Large | 655 (76%) |
| Very large | 155 (18%) |
| **Operative characteristics** |  |
| “Composite repair”^b^, N (%) |  |
| Present | 531 (61.6%) |
| Absent | 331 (38.4%) |
| Esophageal length^c^ |  |
| <2cm | 97 (12%) |
| >2cm | 709 (88%) |
| Anterior crural repair^d^ |  |
| Present | 704 (81.7%) |
| Absent | 158 (18.3%) |
| Total crural sutures, median (IQR) | 4 (3-4) |
| Anterior crural sutures | 1 (1-2) |
| Posterior crural sutures | 2 (2-3) |
| Hiatus closure under tension, N (%) | 83 (9.6%) |
|  |  |

^a^Percentage in mediastinum

^b^Incorporated 360° fundoplication with esophagopexy and cardiopexy to right crus

^c^Intra-abdominal oesophageal length

^d^At least one anterior crural suture used

BMI, Body mass index; ASA, American Society of Anesthesiologists; SD, Standard deviation; IQR, Interquartile range
